# Evidence to shared genetic correlation of ischemic stroke and intracerebral hemorrhage and cardiovascular related traits

**DOI:** 10.1371/journal.pone.0320479

**Published:** 2025-04-23

**Authors:** Wei He, Jiajia Shi, Yiming Qian, Tao Fan, Xuehong Cai, Haochang Li, Peng Huang, Qin Shi

**Affiliations:** 1 Department of Physical Medicine and Rehabilitation, The Affiliated Jiangyin People’s Hospital of Southeast University Medical College, Wuxi, China; 2 Department of Physical Medicine and Rehabilitation, Kunshan Rehabilitation Hospital, Suzhou, China; 3 Department of Neurology, Jiangyin Hospital Affiliated to Nanjing University of Chinese Medicine, Wuxi, China; 4 Department of Epidemiology, Center for Global Health, School of Public Health, Nanjing Medical University, Nanjing, China; University of Catania, ITALY

## Abstract

**Background:**

Previous studies have demonstrated the genetic basis of stroke and also revealed their genetic correlation with some cardiovascular related diseases or traits at the entire genome, which, however, would not give the answer which regions may mainly account for the genetic overlap. This study aims to identify specific genetic loci that contribute to the shared genetic basis between ischemic stroke subtypes and common cardiovascular traits.

**Methods:**

We used Local Analysis of [co]Variant Annotation (LAVA), a recent developed local genetic correlation method, to perform a system local genetic correlation analysis on GWAS summary data of two major subtypes of stroke, including any ischemic stroke (AIS) and intracerebral hemorrhage (ICH), and ten common cardiovascular related diseases or traits (CRTs). We further used colocalization analysis to explore potential shared causal genes in loci with significant local genetic correlation. In addition, we also performed Transcriptome-wide association (TWAS) analysis and fine-mapping for each phenotype to functionally annotate significant loci.

**Results:**

LAVA analysis identified a total of 3 significant local genetic correlations (Bonferroni-adjusted *P* <  0.05) across 3 chromosomes between AIS and systolic blood pressure (SBP), AIS and hypertension (HT), and ICH and body mass index (BMI), among which locus 7.24 explicated to harbor a shared causal variant for AIS and SBP. *TWIST1* in locus 7.24 was defined to be nominally associated with SBP, but not for AIS. Fine-mapping analysis also only identified *TWIST1* a credible causal gene for BMI.

**Conclusions:**

Our study revealed the local genetic correlations between two stroke subtypes and ten common CRTs. Gene-level analyses indicated that biological explanations underlying these identified local genetic correlations may existed elsewhere beyond a common pattern of genetic-gene expression regulation.

## Introduction

Stroke is still a leading cause of both disability and death worldwide, and a major contributor to cognitive decline and dementia, especially in middle- and low-income countries [1,2]. The primary types of stroke are ischemic stroke (IS) and hemorrhagic stroke (e.g., intracerebral hemorrhage [ICH] and subarachnoid hemorrhage [SAH]) [3]. Considering the mechanism of stroke, emerging epidemiological studies have investigated the comorbidity between stroke and cardiovascular-related diseases or traits (CRTs), such as blood cholesterol, body mass index (BMI), atrial fibrillation (AF), and hypertension (HT) [4–6]. Beyond phenotypic comorbidity, the genetic correlation, a crucial aspect of comorbidity, is useful in the mechanism of stroke and other complex diseases [7]. Genome-wide association studies (GWAS), defining numbers of risk loci, provided an opportunity to detect genetic architecture of both stroke and CRTs [8–11]. Given the shared pathological features of stroke and CRTs affecting blood vessels [12], understanding these associations is crucial, as it may reveal shared pathophysiological mechanisms that contribute to both stroke and cardiovascular diseases, thereby informing preventive strategies [13].

Although previous studies have estimated genetic correlations (*r*_*g*_) between stroke and CRTs, they primarily employed global *r*_*g*_ approaches [14–19]. Local *r*_*g*_ in the absence of any global correlation may be undetected. Recently developed methods for assessing local genetic correlations allow for a more nuanced understanding of the shared genetic architecture among complex diseases [19–22]. On the other hand, the correlated regions provide more evidence for the common genes defined by transcriptome-wide association (TWAS). We aim to explore the specific genetic loci shared between two stroke subtypes (AIS and ICH) and seven CRTs, thereby advancing our understanding of their comorbidity.

Here, using accessible GWAS summary statistics, we performed a systemical analysis for the comorbidities between two stroke subtypes, including IS and ICH, and ten CRTs. First, we employed Local Analysis of [co]Variant Annotation (LAVA) [19], to perform a system local genetic correlation analysis. Different to the genome-wide genetic correlation, LAVA employs singular value decomposition to estimate the variance of SNPs after partitioning the genome into semi-independent LD blocks [19]. It enables us to pinpoint shared genetic variants and potential functional genes, facilitating a deeper understanding of the biological mechanisms underlying complex diseases. Secondly, we used colocalization analysis to explore whether the identified loci with significant local genetic correlation contained shared causal genes for both phenotypes. Finally, we performed TWAS analysis and fine-mapping for each phenotype to functionally annotate significant loci.

## Methods and materials

### GWAS summary statistics

We downloaded GWAS data two major subtypes of stroke, including AIS [10] and ICH [23], and ten CRTs, including total cholesterol (TC) [24], HDL [24], low density lipoprotein cholesterol (LDL) [24], logarithm of triglycerides (logTG) [24], BMI [25]; systolic blood pressure (SBP) [26], diastolic blood pressure (DBP) [26], AF [27], HT [28], and familial combined hyperlipidemia (FHL) [29]. Twelve GWASs adopted including from 3,026 to 1,320,016 European individuals. We summarized the detailed demographic information, such as the ratio of sex and heritability, available in the cited studies (S1 Table) [10,23–29]. Specifically, we used the summary statistics for eQTL from GTEx V8, which were available without any application. All cohort data and GWAS resources were approved by relevant ethics committees, and written informed consent was obtained from all participants. We further excluded SNPs with MAF <  0.001, and 5.170 to 11.993 million SNPs were retained for subsequent analysis. A summary of these GWAS summary data is shown in S1 Table. Our data come from the public database where participants have been ethically approved.

### Global genetic correlation analysis

We used linkage disequilibrium score regression (LDSC, v 1.0.1) to estimate (i) the SNP-based heritability (h^2^_SNP_) for each phenotype, (ii) the global genetic correlation (rg) between each phenotype pair, and (iii) the sample overlap [30,31]. We also used 1000 Genomes Project Phase 3 European population as reference panel [32]. Significant SNP-based heritability was defined as with z score >  2, while significant global genetic correlation was defined as with Bonferroni-adjusted *P value* <  0.05.

### Local genetic correlation analysis

We used *LAVA* package (v 0.1.0) to perform local genetic correlation analysis between phenotype pairs [19]. In brief, given the summary statistic of two phenotypes and a locus defined by genomic coordinates or a list of SNPs, *LAVA* can estimate the standard bivariate local rg between the two phenotypes at the defined locus, while accounting for known or estimated sample overlap [19]. In specific, we leveraged the sample overlap estimated by LDSC, and used the predefined loci file by LAVA, which contained 2495 semi-independent LD blocks with a minimum block size of 2500 base pairs (https://github.com/josefin-werme/LAVA/tree/main/support_data) [19,33]. We used *run.univ.bivar* function to perform univariate and bivariate test sequentially. *P value* threshold for the univariate test was set to 0.05/2495 to account for the number of loci tested, and bivariate test was then performed only for the phenotypes that reach the desired univariate significance threshold. Significant bivariate loci were defined as those with Bonferroni-adjusted *P value* <  0.05. Nominal bivariate loci were defined as those with unadjusted *P value* <  0.05, which was used to the following analytic procedures.

### Colocalization analysis

We used *coloc* package (v 5.2.2) to perform Approximate Bayes Factor (ABF) colocalization analysis [34]. In brief, for each nominal bivariate locus detected by LAVA, we used the *coloc.abf* function to assessed the posterior probability that the two phenotypes share same (PPH_4_) or different (PPH_3_) causal variants in this locus [34]. We set the prior probabilities as recommended: (1) 1E-4 for a variant associated with either trait; (2) 1E-5 for a variant associated with both traits; (3) a posterior probability larger than 0.5 was considered as evidence for colocalization.

### TWAS

We performed tissue-specific TWAS analysis using functional summary-based expression imputation algorithm (FUSION) to identify genetic-predicted gene expression associated with each phenotype [35]. We used the tissue-specific predictive models pre-computed on GTEx V8 EUR individuals by the Mancuso lab as TWAS weights. In specific, we downloaded those on 13 different tissues, including eight brain tissues, two heart tissues and one on whole blood, from http://gusevlab.org/projects/fusion/#gtex-v8-multi-tissue-expression. Each weight file contains models for top eQTL, elastic net [36], Sum of Single Effects (SuSiE) [37], and least absolute shrinkage and selection operator (LASSO) [38,39]. For each genetic-predicted gene expression-phenotype pair, FUSION then imputed GWAS Z-scores using the IMPG algorithm; estimated the association statistic, and reported the results of the model with best performance. For each phenotype, significant genes were defined as those with Bonferroni-adjusted *P value* <  0.05.

### Functional analysis of bivariate loci

We used Fine-mapping Of CaUsal gene Sets (FOCUS) to prioritize causal genes within each significant bivariate loci [40]. We downloaded the recommended multi-tissue gene expression weights (https://github.com/bogdanlab/focus/wiki), which combined GTEx V7 weights from PrediXcan with METSIM, NTR, YFS, and CMC weights from FUSION together. FOCUS then calculated the posterior inclusion probability (PIP) for each gene and output a credible set of genes to explain observed genomic risk. Genes with PIP >  0.5 were defined as causal ones.

## Results

### Global and local genetic correlations

**Fig 1:**
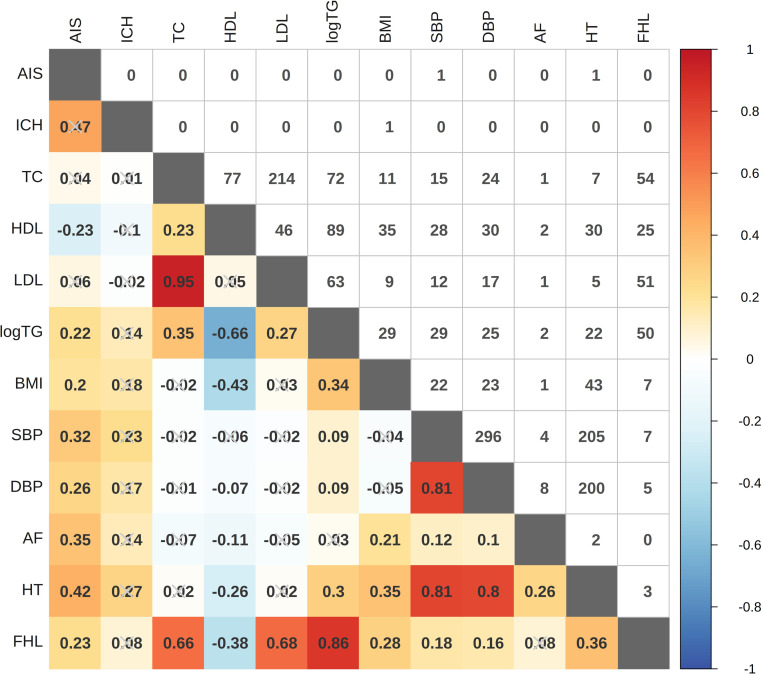
LDSC and LAVA correlations across 12 diseases or traits. The lower triangle displays the global (LDSC) genetic correlations for trait pairs while the upper triangle displays the number of significant bivariate local (LAVA) genetic correlations for each trait pair. Abbreviations: AIS: any ischemic stroke; ICH: intracerebral hemorrhage; TC: total cholesterol; HDL: high density lipoprotein cholesterol; LDL: low density lipoprotein cholesterol; logTG: logarithm of triglycerides; BMI: body mass index; SBP: systolic blood pressure; DBP: diastolic blood pressure; AF: atrial fibrillation; HT: hypertension; FHL: familial combined hyperlipidemia.

We first explored the genetic correlation among the two stroke subtypes and ten CRTs. The results showed that all phenotypes exhibited significant SNP-based heritability, among which the heritability of AIS and ICH were 0.0135 (Z score =  9.6429) and 0.3959 (Z score =  2.6570), respectively (S1 Table). Seven CRTs, including logTG, BMI, SBP, DBP, AF, HT, and FHL, exhibited significantly positive global genetic correlation with AIS, while no CRT showed significant correlation with ICH (S2 Table and Fig 1). We further detected three significant local genetic correlations, which are between AIS and SBP (r =  0.7480, *P value* =  1.62 × 10^-8^, *P*_*adj*_ =  1.42 × 10^-4^), AIS and HT (r =  0.8099, *P value* =  2.66 × 10^-6^, *P*_*adj*_ =  2.35 × 10^-2^), and ICH and BMI (r =  0.8067, *P value* =  1.75 × 10^-6^, *P*_*adj*_ =  1.54 × 10^-2^), respectively (S3 Table and Fig 1). In addition, 124 local genetic correlations showed only nominal significance (unadjusted *P value* <  0.05), of which 40 and 22 are on BMI and DBP, respectively (S3 Table and Fig 1). Of note, 11 of the nominally significant local genetic correlations showed discordant direction with those of corresponding global correlation that showed statistical significance (S2 and S3 Tables).

### Colocalization analysis

**Fig 2:**
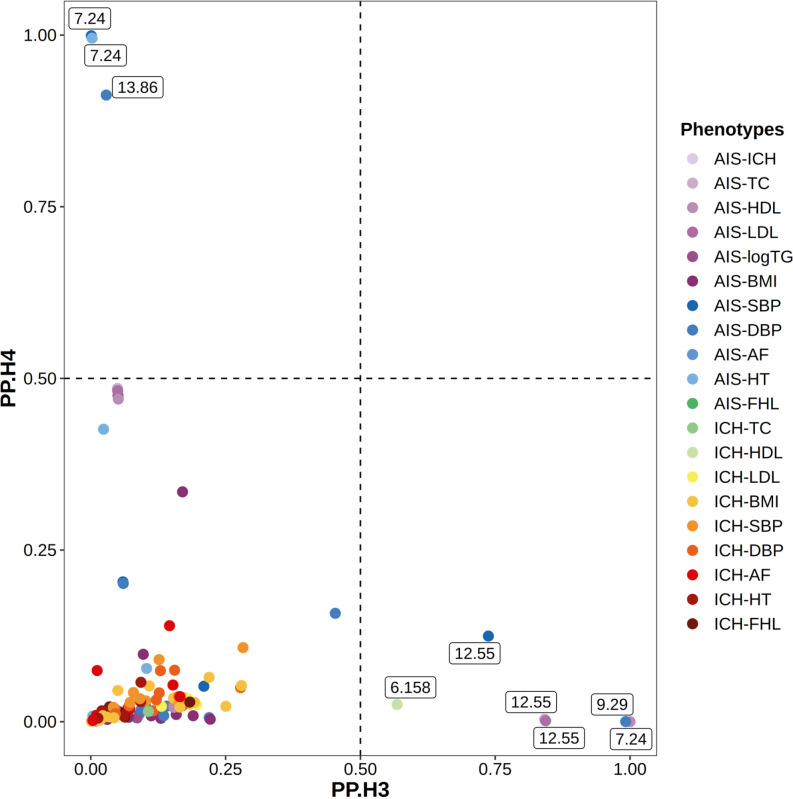
Colocalization analysis of significant bivariate loci. Abbreviations: AIS: any ischemic stroke; ICH: intracerebral hemorrhage; TC: total cholesterol; HDL: high density lipoprotein cholesterol; LDL: low density lipoprotein cholesterol; logTG: logarithm of triglycerides; BMI: body mass index; SBP: systolic blood pressure; DBP: diastolic blood pressure; AF: atrial fibrillation; HT: hypertension; FHL: familial combined hyperlipidemia; PP, posterior probability.

We then performed colocalization to evaluate the posterior probabilities that at least nominally significant bivariate loci comprises independent (H_3_) or shared casual variant (H_4_) for two corresponding phenotypes. The results showed that one of the three significant bivariate loci contained a shared causal variant for AIS and SBP (locus 7.24 with PPH_4_ =  0.999), and two nominal bivariate loci also contained a shared causal variant, among which one is between AIS and HT (locus 7.24 with PPH_4_ =  0.996), and another is between AIS and DBP (locus 13.86 with PPH_4_ =  0.913) (S4 Table and Fig 2). In addition, six nominally significant local genetic correlations indicated potential independent causal variant (with PPH_3_ >  0.5), among which five were on AIS and the highest is between AIS and HDL (locus 7.24 with PPH_3_ =  1.000) (S4 Table and Table 1).

**Table 1:** The summary of pleiotropic loci (PPH3/PPH4 >  0.5).

Locus	Phenotype1	Phenotype2	LAVA.P	PP.H3	PP.H4	TWAS top gene of Phenotype1	Top TWAS.P of Phenotype1	PIP of Phenotype1	TWAS top gene of Phenotype2	Top TWAS.P of Phenotype2	PIP of Phenotype2
7.24	SBP	AIS	1.62E-08	0.001	0.999	TWIST1	0.031	0.149	TWIST1	0.003	0.136
7.24	HT	AIS	1.35E-05	0.002	0.996	SNX13	0.261	0.014	TWIST1	0.003	0.136
13.86	DBP	AIS	0.009	0.029	0.913	LINC00346	0.048	0.022	ANKRD10	0.017	0.005
7.24	HDL	AIS	4.66E-04	1.000	0	SNX13	2.56E-14	0.035	TWIST1	0.003	0.136
9.29	DBP	AIS	0.048	0.992	0	MTAP	1.73E-10	0.024	CDKN2A	9.12E-06	0.015
12.55	LDL	AIS	0.015	0.844	0.001	PIP4K2C	2.58E-07	0.072	TAC3	0.015	0.012
12.55	TC	AIS	0.029	0.842	0.004	TAC3	2.72E-07	0.006	TAC3	0.015	0.012
12.55	SBP	AIS	0.003	0.737	0.125	ARHGEF25	2.64E-09	0.047	TAC3	0.015	0.012
6.158	HDL	ICH	0.11	0.568	0.025	SLC22A3	2.19E-15	0.086	RP11-288H12.3	0.002	0.001

### TWAS

**Fig 3:**
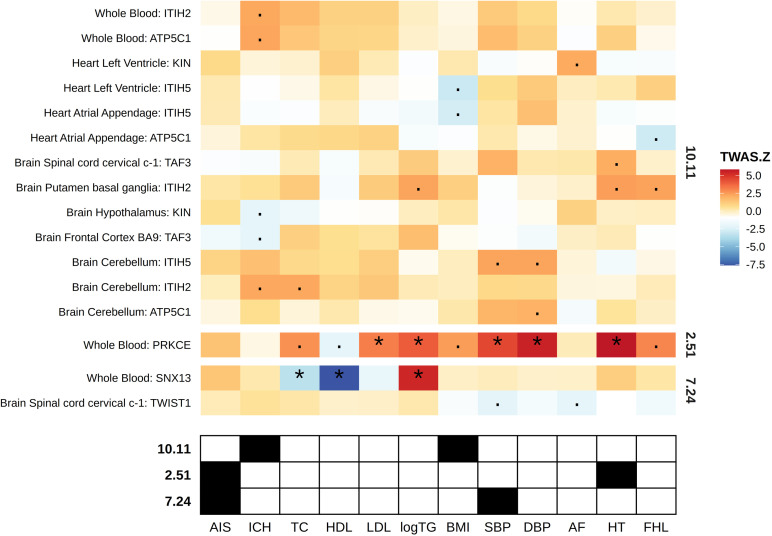
Heatmap indicating concordance of LAVA and TWAS results. The upper heatmap denotes TWAS FUSION z scores for genes within significant LAVA bivariate loci, with “*” indicating significant genes in TWAS analysis (adjusted *P value* <  0.05), and “·” indicating nominally significant genes in TWAS analysis (unadjusted *P value* <  0.05). The lower annotation grid denotes the significance of bivariate phenotype pairs, with black indicating significant pair. Abbreviations: AIS: any ischemic stroke; ICH: intracerebral hemorrhage; TC: total cholesterol; HDL: high density lipoprotein cholesterol; LDL: low density lipoprotein cholesterol; logTG: logarithm of triglycerides; BMI: body mass index; SBP: systolic blood pressure; DBP: diastolic blood pressure; AF: atrial fibrillation; HT: hypertension; FHL: familial combined hyperlipidemia.

In tissue-specific TWAS analysis for each phenotype, we mainly focused on those within significant bivariate loci. *PRKCE* in locus 2.51, a significant bivariate locus for AIS and HT, showed positive association with HT in whole blood (Z score of TWAS =  5.83, and Bonferroni-adjusted *P value* =  1.717E-10), but not with AIS. In addition, *TWIST* in locus 7.24, a significant bivariate locus for AIS and SBP, showed nominally negative association with SBP in brain spinal cord cervical c-1 (Z score =  -2.17, and unadjusted *P value* =  2.99E-02), but not with AIS (S5 and S6 Tables and Fig 3). However, we also detected significant genes in different tissues for nominally significant bivariate loci. For example, SNX13 in locus 7.24 for AIS and HDL showed negative association with HDL in whole blood (Z score of TWAS =  -7.62, and Bonferroni-adjusted *P value* =  8.19E-13) (Table 1).

### Concordance of genetic and gene-based correlations

All three significant bivariate loci identified by LAVA showed relative concordant direction with the global genetic correlations of the corresponding phenotype pair identified by LDSC (S7 Table). We also evaluated the Pearson’s correlation of TWAS statistics Z of genes within a given bivariate locus for the corresponding phenotype pair. As only one and two genes located in locus 2.51 and 7.24, we only assessed the concordance of global and local genetic correlations with the defined TWAS correlation for locus 10.11. However, different from the positive correlation defined by LAVA and LDSC, we did not find any evidence of correlation (S7 Table).

### Gene prioritization

Finally, we performed fine-mapping to prioritize gene-phenotype associations within each bivariate locus. Unexpectedly, only *TWIST1* in locus 7.24 showed to be a credible causal gene of BMI in nerve tibial (PIP =  0.566), although it was also the gene with the highest PIP for both SBP (PIP =  0.149) and AIS (PIP =  0.136) in artery tibial (S8 Table).

## Discussion

In this study, we performed local genetic correlation analysis on two stroke subtypes and ten CRTs. We identified a total of three significant bivariate loci on AIS or ICH. Of note, we also found 11 nominal bivariate loci showed discordant direction with those of corresponding global correlation, indicating that a potential heterogeneity across internal regions existed even between phenotype pairs that exhibited significantly global genetic correlation, and underlined the importance of local genetic correlation analysis in exploring shared genetic basis. Interestingly, our findings were consistent with the previous studies. For example, Laura *et al.* found that the genetic architecture of stroke risk is correlated with that of HT [16]. The primary reason is that they overlap in multiple biological mechanisms, including vascular health, coagulation function, inflammatory response, and metabolic disorders. Different stroke subtypes may act through these common genetic risk factors, but they may also lead to different types of strokes due to distinct pathological processes. Additionally, the interaction between genetics and the environment, as well as the effects of epigenetics, may produce similar genetic effects across various subtypes. Therefore, the genetic susceptibility to stroke is multifaceted, and there is a high degree of genetic overlap between different subtypes.

The locus 7.24 was defined to harbor a significant local genetic correlation for AIS and SBP, and two nominal local genetic correlations for AIS and HDL and AIS and HT. Using colocalization analysis, we found two phenotype pairs (i.e., AIS and SBP and AIS and HT) had a shared causal variant in the locus 7.24, while AIS and HDL may have an independent causal variant in this locus. These results suggested this locus an important contributor to the shared basic of AIS and CRTs. *SNX13* and *TWIST1* are two protein coding genes known to be within locus 7.24 or overlap the boundary of this locus. *SNX13* encodes a protein containing phox-homology domain and the regulator of G protein signaling (RGS) domain, belonging to both the sorting nexin (SNX) and RGS protein families [41]. *SNX13* has been demonstrated to play a potential role in inter-organelle communication and lipid metabolism, and may be involved in the cardiac performance and pancreatic islet function [41–44]. *TWIST1* encodes Twist family BHLH transcription factor 1, and was reported as hypermethylated and overexpressed in multiple human cancers, including lung cancer, prostate cancer, and breast cancer [45–47]. Recent studies also found the contribution of *TWIST1* in vascular diseases like pulmonary hypertension, which may be due to promoting the proliferation of smooth muscle cell [48–50]. In our TWAS analysis, although the genetic predicted expression of *SNX13* and *TWIST1* were found to be significantly associated with HDL and nominally associated with SBP, respectively, we did not identify their association with AIS. Our fine-mapping analysis also only identified *TWIST1* a credible causal gene for BMI. These results may not support common genetic-gene expression regulation patterns underlying the shared genetic basis among these stroke subtypes and CRTs.

To our best knowledge, our study is the first to take into consideration the local genetic correlation between stroke and CRTs, enabling it to provide some additional clues beyond global genetic correlation analysis for understanding the shared genetic basis between stoke and CRTs. To maximize statistical power and representativeness, we included GWAS summary data with nearly the largest sample size publicly available. In addition, for identified loci with even only nominal significance, we employed a series of validation tools, including colocalization, TWAS, and fine-mapping, to further explore the potential key genetic variants or genes that may drive the local genetical correlation. But several limitations of our study warrant consideration. First, our analysis is based on GWAS data predominantly from individuals of European ancestry. It may restrict the generalizability of our findings to other ethnic groups and bring statistical bias in the observed genetic correlations [14,51]. Second, because of the low prevalence and distinct pathophysiological mechanisms, we primarily focused on AIS and ICH, rather than SAH. The low case number in the GWAS summary statistics make it difficult to analyze the genetic associations [52]. Third, due to the presence of different subtypes of stroke, there may be heterogeneity within our study population, which could lead to incomplete results. Therefore, future research should consider these differences in the design, data analysis, and interpretation of results, in order to draw more accurate, comprehensive, and meaningful conclusions [53,54]. Finally, with our knowledge, cardiovascular diseases might be more closely related to ICH. But SAH is commonly seen in conditions such as aneurysms and arteriovenous malformations. Third, the extensive multiple testing we made may also decrease the statistical power in local genetic correlation. To alleviate this issue, we also pay considerable attention to those nominally significant loci in our analysis. The integration of genetic insights with clinical data could enhance our understanding of stroke mechanisms and guide personalized interventions.

In summary, we explored the shared genetic basis of two stroke subtypes and ten CRTs in a series of semi-independent genomic loci via local genetic correlation analysis. We found a strong stroke-CRT genetic correlation at a total three loci, and also found a potential heterogeneity across internal regions between phenotype pairs showing significant global correlation. Gene-level analyses did not identify potential pleiotropic genes, indicating that the biological explanations underlying these identified local genetic correlations may exist elsewhere beyond a common pattern of genetic-gene expression regulation, and further exploration is needed.

## Supporting information

S1 Table Phenotype and sample information. (XLSX)

S2 Table LDSC genetic correlations across 12 diseases or traits. (XLSX)

S3 Table LAVA local genetic correlations across 12 diseases or traits. (XLSX)

S4 Table COLOC results for loci with a nominally significant local genetic correlation. (XLSX)

S5 Table TWAS FUSION results for 12 phenotypes. (XLSX)

S6 Table TWAS FUSION Z scores for genes within the bivariate loci. (XLSX)

S7 Table Comparison of LDSC, LAVA, and TWAS correlations. (XLSX)

S8 Table FOCUS fine-mapping results. (XLSX)
